# The Impact of Climate Vulnerability on Cancer Incidence Among U.S. Women

**DOI:** 10.7759/cureus.102478

**Published:** 2026-01-28

**Authors:** Caitlin Johnson, Cheng-I Liao, Ruolin Lorraine Jiang, Nathan Tran, Kim Duong, Amandeep Mann-Grewal, Daniel S Kapp, John K Chan

**Affiliations:** 1 Gynecologic Oncology, California Pacific Medical Center Research Institute, San Francisco, USA; 2 Obstetrics and Gynecology, Kaohsiung Veterans General Hospital, Kaohsiung, TWN; 3 Surgery, University of California, San Francisco, San Francisco, USA; 4 Osteopathic Medicine, Western University of Health Sciences, Pomona, USA; 5 Research, Center for Health Systems Research, Sutter Health, Walnut Creek, USA; 6 Radiation Oncology, Stanford University School of Medicine, Stanford, USA

**Keywords:** breast cancer, breast cancer incidence, cancer incidence, cancer incidence rates, climate change, climate change and its effect on life and health, climate vulnerability, lung cancer, melanoma

## Abstract

Background and aim

While climate change and its impacts have intensified in the U.S. over the past decade, the relationship between cancer incidence and regional climate vulnerability among women, particularly racial and ethnic minorities, remains largely underexplored. Climate-related environmental exposures, such as extreme weather events, air pollution, and ultraviolet radiation, are linked to risk factors for melanoma, lung, and breast cancers, yet national-level assessments across regions with differing climate impacts are limited. Understanding how these patterns vary geographically and among populations is critical for identifying emerging health disparities. This study aimed to examine the association between cancer incidence trends and state-level climate-impact categories among U.S. women, with additional stratification by racial and ethnic groups.

Methods

U.S. states were categorized into three climate impact levels (high, moderate, and low) based on climate data from federal, insurance, and nonprofit reports. We used the United States Cancer Statistics data to identify 6,728,838 climate change-associated cancers (cutaneous melanoma, lung, and breast) diagnosed from 2001 to 2019. Statistical analyses were performed to examine incidence trends for these cancers.

Results

High climate impact states experienced larger increases in cutaneous melanoma (average annual percent change (AAPC) 2.82%; p = 0.002), lung cancer (AAPC -0.94%; p < 0.001), and breast cancer incidence (AAPC 0.34%; p = 0.008) compared to low climate impact states (cutaneous melanoma: 2.24%, p < 0.001; lung cancer: AAPC -1.34%, p < 0.001; breast cancer: -0.31%, p = 0.03). Analysis by race/ethnicity showed that breast cancer incidence in high-impact regions increased among Black (AAPC 2.29%; p = 0.001), Hispanic (AAPC 2.21%; p = 0.003), and Asian women (AAPC 0.90%; p < 0.001) but decreased among White women (AAPC -0.52%; p < 0.001).

Conclusions

Our results indicate that cancer incidence patterns among U.S. women vary across climate impact categories and racial groups, suggesting that climate vulnerability may contribute to persistent disparities in melanoma, lung, and breast cancer trends. Women living in higher climate-impact regions experienced less favorable incidence patterns, particularly among racial and ethnic minority populations. Although these findings are ecological in nature, they highlight population-level associations that warrant further investigation using individual-level exposure data and accounting for socioeconomic and healthcare-related factors. As climate-related hazards continue to intensify, these results underscore the importance of integrating climate vulnerability into cancer surveillance, prevention strategies, and policies aimed at reducing environmental and health inequities.

## Introduction

Climate change refers to the long-term shift in average weather patterns in a given location, explicitly focusing on changes in the type, frequency, duration, and intensity of weather events [1]. In particular, rising atmospheric temperatures are associated with an increased frequency of extreme weather events, such as hurricanes, ozone depletion, and wildfires, which can affect oncological healthcare outcomes through multiple etiological pathways [1]. These climate-related environmental changes influence human health through several mechanisms, including increased UV radiation exposure, deteriorating air quality, and disruptions to essential healthcare services [2].

Cancer is the second most common cause of death among women [2]. Breast, lung, and skin (melanoma) cancers are considered climate change-associated due to the growing risks linked to the deleterious impacts of climate change [2]. Long-term exposure to wildfire-related fine particulate matter (≤2.5 μm in diameter) with high oxidative potential is associated with increased lung cancer risk [3,4]. Breast cancer risk is linked to increasing radiation exposure and decreasing water vapor in lower-altitude regions during extreme weather events [5]. Additionally, reduced access to healthcare caused by adverse weather events contributes to elevated cancer risks. However, prior regional studies do not adequately address changes in cancer incidence and the impacts of climate change across the U.S. over the past two decades.

Severe health impacts from climate change have been identified in previous studies [2]. Yet, there is limited focus on cancer incidence within marginalized populations, with women disproportionately affected compared to men [6,7]. Although lung cancer continues to have a high mortality rate, its incidence is decreasing among men, in contrast to an increasing trend among women in recent years [7]. Moreover, while climate change-associated extreme weather events occur nationwide, racial and ethnic minority groups are significantly more vulnerable than the general population [8,9]. Historical redlining has also contributed to present-day unequal exposure to climate risks, including high temperatures and flood hazards, with low-income families often residing in communities affected by these past policies [10,11].

Examining the effects of climate change on cancer incidence from the perspective of gender and race has been largely overlooked in prior studies [12,13]. Despite evidence linking climate-related exposures to specific cancer risk factors, there is limited national-level research assessing how cancer incidence trends differ across regions with varying climate vulnerability. Given these gaps, the present analysis provides a large-scale assessment of how climate vulnerability correlates with melanoma, lung, and breast cancer incidence over nearly two decades among U.S. women.

This article was previously posted to the Research Square preprint server on August 5, 2024 [14].

## Materials and methods

This study employed a retrospective ecological design to examine associations between state-level climate impact categories and cancer incidence trends among U.S. women. The unit of analysis was the U.S. state, with cancer incidence data aggregated at the state level and compared across climate impact categories to characterize population-level patterns across geographic regions with varying climate vulnerabilities.

Study setting and population

The study population comprised all women diagnosed with breast cancer, lung and bronchus cancer, or cutaneous melanoma in the U.S. from 2001 to 2019, as captured by the United States Cancer Statistics (USCS) database [15]. The USCS database integrates data from the National Program of Cancer Registries (NPCR) and the Surveillance, Epidemiology, and End Results (SEER) Program, covering approximately 98% of the U.S. population. Race was categorized according to USCS guidelines: Hispanic, non-Hispanic Asian/Pacific Islander, non-Hispanic Black, non-Hispanic White, and other/unknown. Cancer stage was categorized as localized, regional, distant, and unknown/other. Age was categorized as 0-19, 20-44, and ≥45 years. All incident cases of breast cancer, lung and bronchus cancer, and cutaneous melanoma diagnosed among women between January 1, 2001, and December 31, 2019, with valid state-level geographic identifiers, were included. Nevada and Mississippi were excluded from all analyses because incidence data for these states were not available for the entire study period.

Climate impact categories

U.S. states were categorized into three climate impact categories (high, moderate, and low) using climate change data from 2007 to 2023 obtained from federal reports, insurance companies, and nonprofit organizations [15-20]. Climate change data included extreme weather events (weather or climate events of unusual severity, including wildfires, heatwaves, storms, heavy precipitation, flooding, and drought), climate change index scores, and state resiliency planning [1,21-23]. Sources were included if they reported at least one key climate domain and were published by federal agencies, peer-reviewed organizations, insurance industry analyses, or established nonprofit research organizations, but were excluded if they provided only regional and not state-level data.

To categorize each U.S. state into one of the three climate impact categories, states were ranked on an 11-point scale from -5 to +5 for each data source after merging using state-level identifiers (state name and FIPS code) as the common linkage variable. High climate impact states were defined as those with a greater frequency of extreme weather events, a higher climate change index ranking, and lower climate resiliency planning. Low climate impact states were defined as those with fewer extreme weather events, lower climate change index rankings, and higher climate resiliency planning. The five states with the highest climate impact were ranked from +1 to +5, with +5 assigned to the state with the highest climate impact and +1 to the state with the fifth-highest climate impact. The five states with the lowest climate impact were ranked from -1 to -5, with -5 assigned to the state with the lowest climate impact and -1 to the fifth-lowest. States not in the top or bottom five climate change categories were designated as 0.

Each source contributed independent rankings that were summed to create aggregate climate impact scores, and all data sources received equal weighting to minimize source bias. States ranking within the top 75th percentile were designated as high climate impact states, those within the 25th percentile were designated as low climate impact states, and states between the 25th and 75th percentiles were defined as moderate climate impact states. This approach was informed by and adapted from prior studies’ ranking methods, where continuous vulnerability indices are converted into percentile- or quantile-based categories for descriptive and comparative analyses [24-27].

## Results

Climate impact state scores

**Figure 1:**
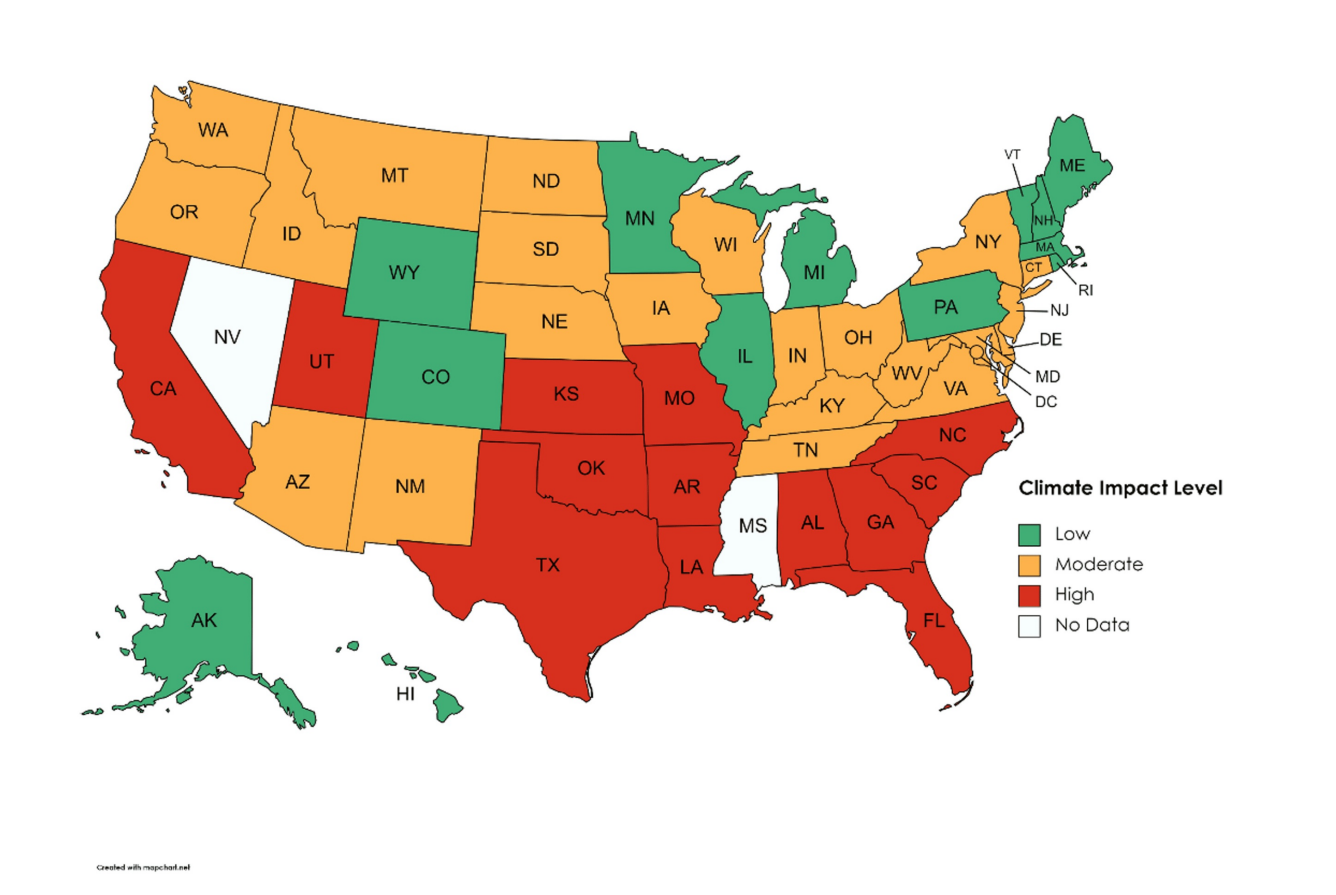
Color-coded map of the U.S. depicting climate impact levels Map created using mapchart.net

The 13 states in the top 75th percentile, classified as high climate impact, were Alabama, Arkansas, California, Florida, Georgia, Kansas, Louisiana, Missouri, North Carolina, Oklahoma, South Carolina, Texas, and Utah. The 13 states in the bottom 25th percentile, classified as low climate impact, were Alaska, Colorado, Hawaii, Illinois, Maine, Massachusetts, Michigan, Minnesota, New Hampshire, Pennsylvania, Rhode Island, Vermont, and Wyoming. The remaining 24 states were considered moderate climate impact regions (Figure 1).

Overall demographics

From 2001 to 2019, there were 6,728,838 incident cases of breast, skin (melanoma), and lung cancers diagnosed in women. Of these, 8.1% were cutaneous melanoma, 28.3% were lung and bronchus cancers, and 63.6% were breast cancers. Regarding race, 80.1% of patients were non-Hispanic White, 9.7% were non-Hispanic Black, 6.1% were Hispanic, 2.9% were Asian, and 1.3% identified as other/unknown. Regarding age, 0.1% were 0-19 years, 9.0% were 20-44 years, and 90.9% were aged 45 years or older. Regarding cancer stage, 52.5% of cases were localized, 25.0% were regional, 16.7% were distant, and 5.8% were unknown.

Cutaneous melanoma

**Figure 2:**
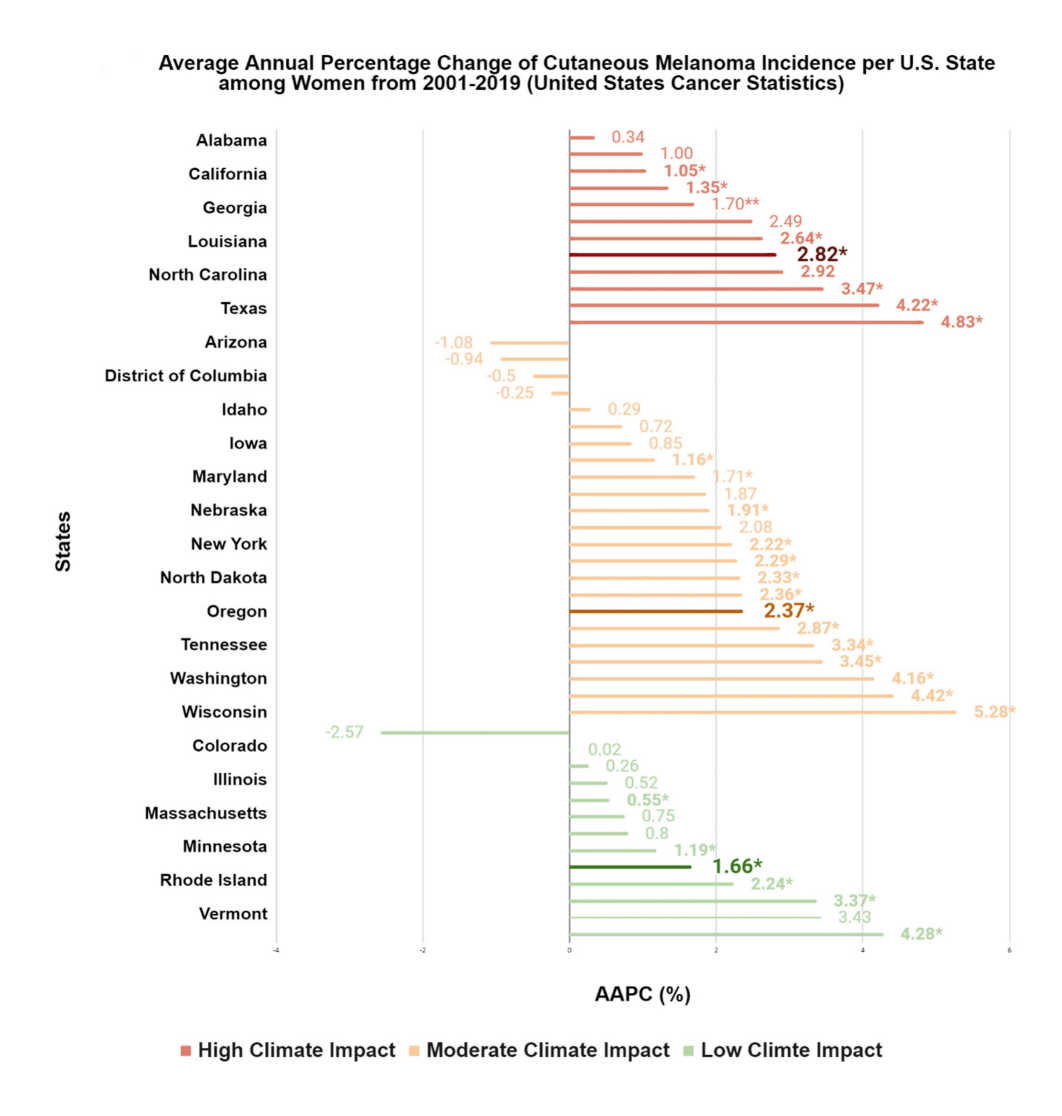
AAPC of cutaneous melanoma incidence per U.S. state among women from 2001 to 2019 This figure depicts the AAPCs for incident cutaneous melanoma for each U.S. state from 2001 to 2019, grouped by climate impact region. SEERStat 8.3.9.2 and the Joinpoint Regression Program 4.9.0.0 were used to calculate cancer incidences and trends per 100,000. AAPCs that are statistically significant (p < 0.05) are indicated by an asterisk (*). The statistically significant median AAPC is bolded for ease of reference. AAPC, average annual percent change Source: United States Cancer Statistics [17]

From 2001 to 2019, cutaneous melanoma incidence increased nationally among women by 1.59% per year, rising from 13.87 to 18.23 per 100,000 (p = 0.008). High climate impact states had, on average, higher average annual percent changes (AAPCs) than moderate and low impact states, indicating steeper incidence increases in more climate-vulnerable regions. Rather than detailing every state individually, these patterns are summarized at the climate impact category level, with state-specific AAPCs provided in Table 1 and Figure 2.

**Table 1:** Cutaneous melanoma, lung and bronchus, and breast cancer incidence by race and climate impact region in the U.S. Nevada and Mississippi were excluded from the analysis because data were not available. Oklahoma did not report cutaneous melanoma data. SEER*Stat 8.3.9.2 and the Joinpoint Regression Program 4.9.0.0 were used to calculate cancer incidences and trends per 100,000. ᵃ Incidences reported per 100,000 ᵇ Even number of statistically significant AAPCs; therefore, two values are presented instead of one AAPC, average annual percent change

Cancer type	Climate impact region by race/ethnicity	AAPCᵃ	2001 incidence	2019 incidence	95% upper CI	95% lower CI	P-value
Cutaneous melanoma	White						
	High	2.99	11.62	16.58	1.14	4.86	0.001
	Moderate	2.85	14.48	22.38	2.14	3.58	<0.001
	Lowᵇ	1.92	17.66	27.66	0.52	3.33	0.011
	Lowᵇ	1.8	18.63	32.1	0.48	3.14	0.01
	Black						
	High	-2.33	1.23	1.04	-4.2	-0.42	0.02
	Moderateᵇ	-2.25	0.66	0.75	-4.34	-0.11	0.041
	Moderateᵇ	-4.02	1.07	0.68	-7.43	-0.48	0.029
	Low	-2.67	0.98	0.6	-4.88	-0.41	0.023
	Hispanic						
	Highᵇ	0.89	4.27	5.24	0.1	1.69	0.029
	Highᵇ	-5.77	13.72	4.6	-11.16	-0.05	0.048
	Moderate	-1.95	3.57	2.65	-3.65	-0.22	0.03
	Lowᵇ	2.96	1.17	5.59	1.05	4.9	0.004
	Lowᵇ	2.04	4.99	7.73	0.34	3.77	0.021
	Asian						
	Highᵇ	0.1	1.15	1.33	-1.55	1.79	0.9
	Highᵇ	-3.1	2.36	0.75	-7.33	1.32	0.2
	Moderate^b^	0.18	0.5	1.33	-2.37	2.8	0.9
	Moderateᵇ	-1.09	0.66	0.57	-7.33	5.57	0.7
	Low	-2.88	1.57	1.64	-5.26	-0.44	0.024
Lung and bronchus	White						
	High	-0.91	64.81	56.88	-1.32	-0.5	<0.001
	Moderate	-1.34	55.66	41.99	-1.87	-0.8	<0.001
	Lowᵇ	-0.87	43.95	46.44	-11.69	-0.44	0.041
	Lowᵇ	-1.4	47.89	38.13	-1.74	-1.06	<0.001
	Black						
	High	-0.82	50.33	45.25	-1.39	-0.26	0.004
	Moderate	-1.36	53.11	40.76	-1.87	-0.85	<0.001
	Low	-1.34	43.9	41.5	-2.39	-0.27	0.017
	Hispanic						
	High	-2.24	26.5	18.32	-2.61	-1.86	<0.001
	Moderateᵇ	0.55	27.75	29.62	0.01	1.08	0.046
	Moderateᵇ	-5.99	82.62	42.66	-10.52	-1.23	0.017
	Low	3.53	41.71	88.31	0.85	6.29	0.012
	Asian						
	High	-1.51	25.97	19.28	-2.74	-0.27	0.02
	Moderate	2.65	20.55	38.1	1.92	3.39	<0.010
	Lowᵇ	3.6	15.73	29.52	11.43	5.81	0.003
	Lowᵇ	1.56	17.85	33.14	0.42	2.72	<0.001
Breast	White						
	High	-0.52	140.74	129.09	-0.8	-0.24	<0.001
	Moderateᵇ	0.4	118.2	122.8	0.18	0.63	0.002
	Moderateᵇ	0.38	141.65	149.99	0.2	0.57	<0.001
	Low	0.44	143.2	140.99	0.05	0.84	0.03
	Black						
	Highᵇ	0.96	118.55	136.96	0.67	1.26	<0.001
	Highᵇ	0.9	108.52	132.49	0.63	1.17	<0.001
	Moderate	0.8	116.42	132.85	0.23	1.38	0.009
	Low	0.18	116.08	123.97	-0.47	0.83	0.6
	Hispanic						
	High	2.29	88.15	91.7	1.12	3.47	0.001
	Moderateᵇ	1.31	96.31	96.63	0.56	2.07	0.002
	Moderateᵇ	0.92	96.1	113.98	0.36	1.47	0.003
	Lowᵇ	1.16	86.61	104.51	0.6	1.71	<0.001
	Lowᵇ	0.7	97.86	112.34	0.15	1.26	0.016
	Asian						
	Highᵇ	2.33	67.92	94.74	1.31	3.37	<0.001
	Highᵇ	2.21	43.8	89.77	0.83	3.61	0.003
	Moderate	2.08	58.58	83.07	0.35	3.84	0.021
	Lowᵇ	1.16	86.61	104.51	0.6	1.71	<0.001
	Lowᵇ	0.7	97.86	112.34	0.15	1.26	0.016

The impact of climate change on cutaneous melanoma varied among racial groups. White and Hispanic populations experienced annual increases of 2.85% (p < 0.001) and 0.89% (p = 0.03), respectively, while Black and Asian populations experienced annual decreases of -2.33% (p = 0.02) and -2.88% (p = 0.02). These decreases were most pronounced in low climate impact states for Black (-2.67%, p = 0.02) and Asian (-2.88%, p = 0.02) populations. Among White women, melanoma incidence increased by 2.99% (p = 0.001), 2.85% (p < 0.001), and 1.80% (p = 0.01) in high, moderate, and low climate impact states, respectively. Additional details are provided in Table 1.

Lung and bronchus

**Figure 3:**
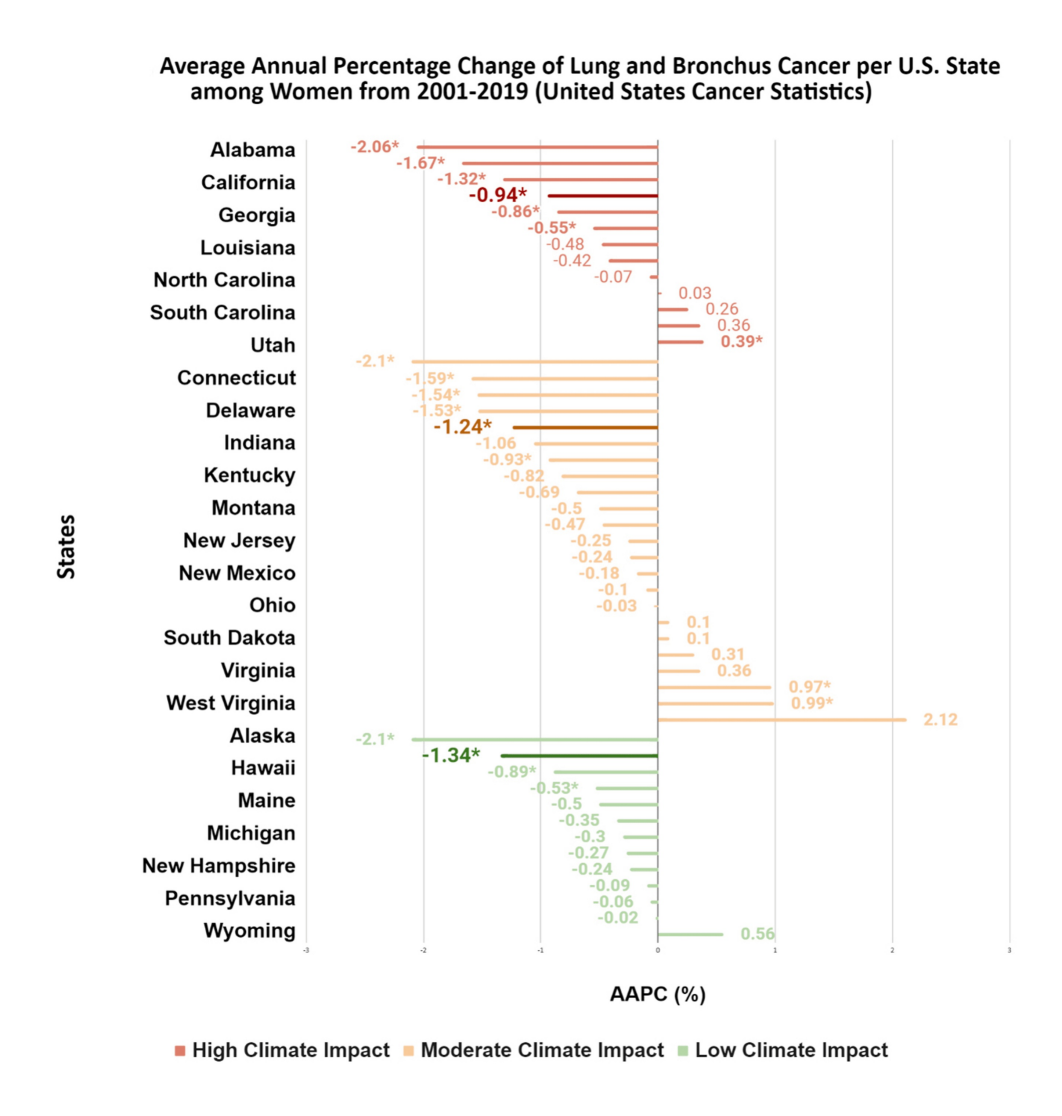
AAPC of lung and bronchus cancer per U.S. state among women from 2001 to 2019 This figure depicts AAPCs for incident lung and bronchus cancer for each U.S. state by climate impact region. SEERStat 8.3.9.2 and the Joinpoint Regression Program 4.9.0.0 were used to calculate cancer incidences and trends per 100,000. AAPCs that are statistically significant (p < 0.05) are indicated by an asterisk (*). The statistically significant median AAPC is bolded for ease of reference. AAPC, average annual percent change Source: United States Cancer Statistics [17]

Overall, lung cancer incidence declined across climate impact categories (-0.73% per year, from 54.97 to 48.12 per 100,000 over the study period; p < 0.001). However, trends varied by state, with some states exhibiting stable or increasing incidence despite the overall downward trend. For distant-stage disease, incidence declines were larger in low (AAPC: -1.94%; p < 0.001) and moderate impact states (AAPC: -1.86%; p < 0.001) compared to high climate impact regions (AAPC: -1.15%; p = 0.01) (Figure 3).

All racial groups experienced declines in lung cancer incidence, with the most pronounced decreases observed in Asian (-1.51%; p = 0.02) and Hispanic (-2.24%; p < 0.001) populations. Decreases were smaller among White (-0.91%; p < 0.001) and Black (-0.82%; p = 0.004) populations in high climate impact states (Table 1).

Breast

**Figure 4:**
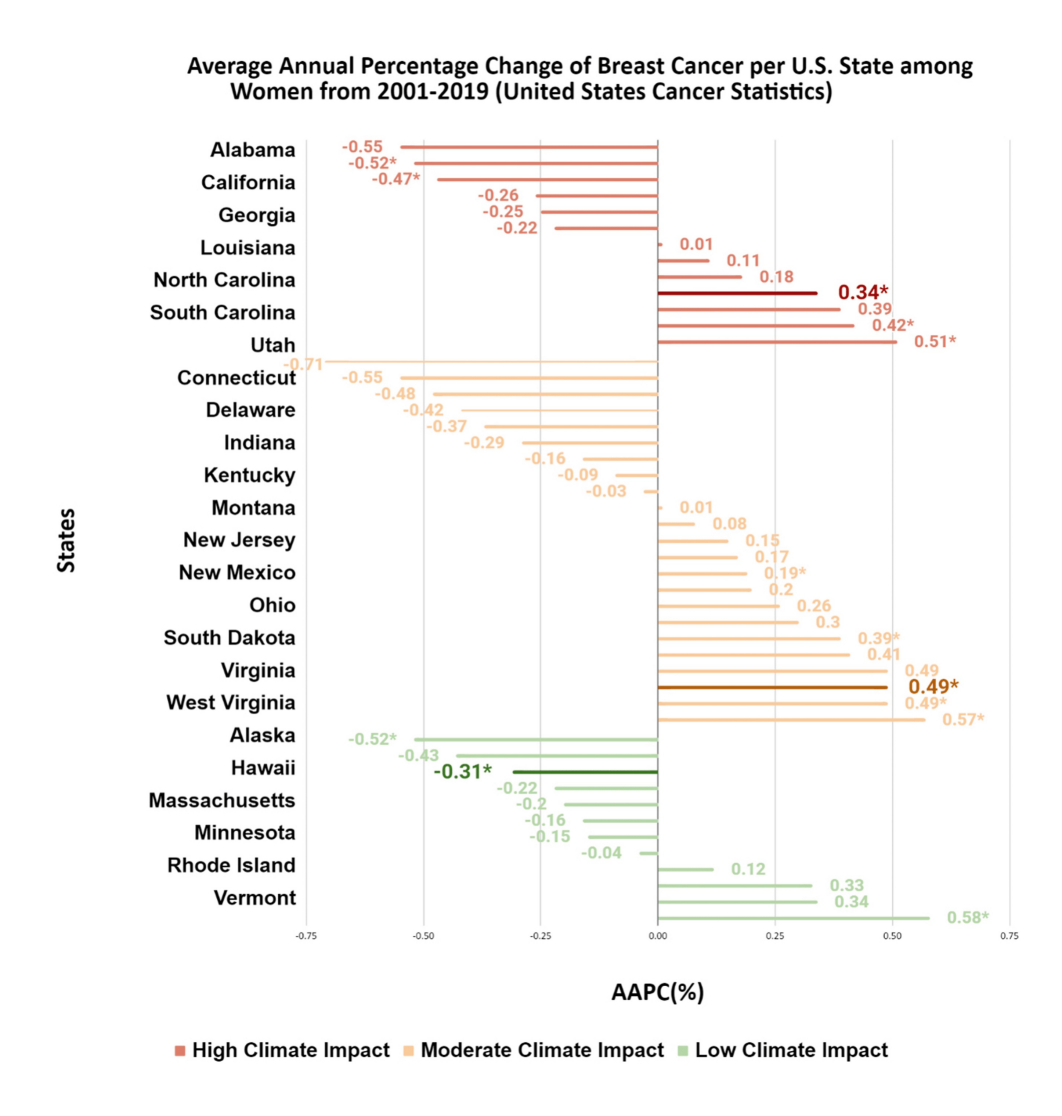
AAPC of breast cancer per U.S. state among women from 2001 to 2019 This figure depicts AAPCs for incident breast cancer for each U.S. state from 2001 to 2019, grouped by climate impact region. SEERStat 8.3.9.2 and the Joinpoint Regression Program 4.9.0.0 were used to calculate cancer incidences and trends per 100,000. AAPCs that are statistically significant (p < 0.05) are indicated by an asterisk (*). The statistically significant median AAPC is bolded for ease of reference. AAPC, average annual percent change Source: United States Cancer Statistics [17]

From 2001 to 2019, breast cancer incidence in the U.S. decreased slightly by -0.12% per year, from 131.97 to 129.68 per 100,000 (p = 0.001). On average, breast cancer incidence in high-impact states increased modestly over the study period, whereas low-impact states showed flatter or declining trends. These averages mask considerable heterogeneity, with some high-impact states experiencing larger increases and others smaller changes (Figure 4).

In high-impact states, breast cancer incidence generally increased among Black, Hispanic, and Asian women, although the magnitude of AAPCs varied by state and was not uniformly elevated across all high-impact states. The clearest pattern was a relative increase in incidence among racial and ethnic minority women compared with White women in these regions. White women experienced annual decreases of -0.52% (p < 0.001), while Black, Hispanic, and Asian women experienced annual increases of 2.29% (p = 0.001), 2.21% (p = 0.003), and 0.90% (p < 0.001), respectively (Table 1).

Additional information on cutaneous melanoma, lung and bronchus, and breast cancer incidence can be found in Table 2, Table 3, Table 4, Table 5, and Table 6.

**Table 2:** Incidence of cutaneous melanoma, lung and bronchus, and breast changes among adult women in the U.S. (2001-2019) Nevada and Mississippi were excluded from the analysis because data were not available. Oklahoma did not report cutaneous melanoma data. SEER*Stat 8.3.9.2 and the Joinpoint Regression Program 4.9.0.0 were used to calculate cancer incidences and trends per 100,000. Statistically significant results are bolded for ease of reference. ᵃ Incidences reported per 100,000 * P < 0.05, ** P < 0.001 AAPC, average annual percent change Source: United States Cancer Statistics [17]

U.S. states	Cutaneous melanoma AAPCᵃ (95% CI)	Lung and bronchus AAPCᵃ (95% CI)	Breast AAPCᵃ (95% CI)
High climate impact			
Alabama	2.49 (-1.91, 7.09)	-0.42 (-0.94, 0.11)	0.34^*^ (0.10, 0.59)
Arkansas	4.83^**^ (4.11, 5.55)	0.39^*^ (0.11, 0.68)	0.01 (-0.80, 0.84)
California	1.05^**^ (0.75, 1.35)	-2.06^**^ (-2.29, -1.82)	-0.52^*^ (-0.99, -0.04)
Florida	1.35^*^ (0.03, 2.69)	-1.32^**^ (-1.55, -1.08)	-0.47^*^ (-0.78, -0.15)
Georgia	1.70^**^ (0.80, 2.60)	-0.55 (-0.89, -0.22)	0.11 (-0.30, 0.53)
Kansas	2.64^**^ (2.12, 3.17)	-0.07 (-0.82, 0.68)	-0.22 (-1.05, 0.61)
Louisiana	2.92^**^ (2.30, 3.55)	-0.86^**^ (-1.15, -0.57)	0.51^*^ (0.26, 0.77)
Missouri	2.82^**^ (1.01, 4.65)	0.26 (-0.24, 0.76)	0.42^*^ (0.16, 0.68)
North Carolina	3.47^**^ (2.04, 4.92)	0.36 (-0.59, 1.32)	0.39 (-0.10, 0.88)
Oklahoma	-	-0.94^**^ (-1.28, -0.60)	-0.55 (-1.22, 0.13)
South Carolina	1.00 (-0.52, 2.54)	-0.48 (-0.97, 0.01)	0.18 (-0.47, 0.84)
Texas	0.34 (-1.04, 1.74)	-1.67^**^ (-2.07, -1.26)	-0.26 (-1.12, 0.62)
Utah	4.22^**^ (3.59, 4.85)	0.03 (-0.57, 0.64)	-0.25 (-1.19, 0.69)
Moderate climate impact			
Arizona	1.87 (-0.23, 4.02)	-1.59^**^ (-2.12, -1.06)	-0.55 (-1.67, 0.59)
Connecticut	-1.08 (-2.25, 0.10)	-0.47 (-1.09, 0.16)	-0.29 (-0.83, 0.26)
District of Columbia	-0.25 (0.41, 4.20)	-1.54^*^ (-2.27, 1.29)	-0.42 (0.04, 1.10)
Delaware	2.29^*^ (-2.05, 1.58)	-0.50 (-2.65, -0.42)	0.57^*^ (-1.29, 0.46)
Idaho	1.91^*^ (0.76, 3.07)	0.31 (-1.26, 1.90)	-0.16 (-1.21, 0.91)
Indiana	0.72 (-2.16, 3.68)	-0.82 (-1.77, 0.14)	-0.37 (-1.12, 0.38)
Iowa	2.33^*^ (0.50, 4.20)	0.36 (-1.55, 2.31)	0.49 (-0.07, 1.05)
Kentucky	2.22^**^ (1.71, 2.73)	0.10 (-0.17, 0.38)	0.15 (-0.24, 0.56)
Maryland	1.16^**^ (0.52, 1.81)	-0.69 (-1.63, 0.25)	0.30 (-0.01, 0.61)
Montana	3.34^**^ (2.70, 3.99)	-1.24^*^ (-2.01, -0.47)	0.41 (-0.12, 0.95)
Nebraska	4.42^**^ (2.49, 6.39)	-0.03 (-0.51, 0.45)	0.01 (-0.69, 0.71)
New Jersey	0.29 (-0.29, 0.87)	-0.93^**^ (-1.23, -0.63)	0.08 (-0.42, 0.60)
New York	2.36^**^ (-2.23, 0.37)	-0.10 (-1.86, -0.26)	0.49*^*^ (-0.17, 0.50)
New Mexico	-0.94 (1.26, 3.48)	-1.06 (-0.41, 0.22)	0.17 (0.30, 0.68)
North Dakota	5.28^*^ (2.24, 8.41)	2.12 (-0.29, 4.59)	0.49* (0.08, 0.91)
Ohio	2.37^**^ (1.59, 3.17)	-0.18 (-0.47, 0.12)	0.19 (-0.15, 0.53)
Oregon	-0.50 (-1.94, 0.95)	-2.10 (-2.86, -1.33)	-0.48 (-1.18, 0.23)
South Dakota	4.16^**^ (2.94, 5.41)	0.97^*^ (0.42, 1.53)	-0.03 (-0.74, 0.68)
Tennessee	3.45^*^ (1.12, 5.83)	0.99^*^ (0.25, 1.74)	0.39^*^ (0.16, 0.61)
Virginia	2.08 (-1.49, 5.78)	-0.24 (-1.00, 0.52)	0.20 (-0.02, 0.42)
Washington	0.85 (-0.50, 2.21)	-1.53^*^ (-1.77, -1.29)	-0.71 (-1.83, 0.42)
West Virginia	1.71^**^ (0.92, 2.50)	-0.25 (-0.73, 0.23)	0.26 (-0.06, 0.57)
Wisconsin	2.87^**^ (1.85, 3.89)	0.10 (-0.16, 0.36)	-0.09 (-0.45, 0.28)
Low climate impact			
Alaska	0.75 (-4.73, 6.54)	-2.10^**^ (-3.02, -1.17)	-0.52^*^ (-0.95, -0.09)
Colorado	0.02 (-0.55, 0.60)	-1.34^**^ (-1.74, -0.94)	-0.20 (-0.79, 0.39)
Hawaii	1.19^*^ (0.22, 2.17)	-0.02 (-1.16, 1.14)	0.33 (-1.47, 2.16)
Illinois	3.37^**^ (2.82, 3.92)	-0.35 (-0.81, 0.12)	0 (-0.46, 0.47)
Maine	2.24^**^ (1.36, 3.12)	-0.09 (-0.36, 0.19)	-0.31^*^ (-0.59, -0.03)
Massachusetts	-2.57 (-5.22, 0.15)	-0.50 (-1.00, 0.01)	0.12 (-0.10, 0.35)
Michigan	0.55^*^ (0.01, 1.08)	-0.53^*^ (-0.98, -0.08)	-0.43 (-0.95, 0.09)
Minnesota	4.28^**^ (3.71, 4.85)	-0.06 (-0.94, 0.82)	-0.15 (-1.00, 0.70)
New Hampshire	0.52 (-0.66, 1.72)	-0.30 (-0.71, 0.11)	0.34 (-0.05, 0.74)
Rhode Island	0.26 (-1.17, 2.80)	-0.27 (-0.97, 0.49)	0.58^**^ (-0.65, 0.33)
Pennsylvania	0.80 (-0.47, 1.00)	-0.24 (-1.63, 1.12)	-0.16 (0.17, 1.00)
Vermont	3.43 (-1.35, 8.44)	0.56 (-0.91, 2.06)	-0.04 (-0.39, 0.31)
Wyoming	1.66^*^ (0.27, 3.06)	-0.89^*^ (-1.68, -0.10)	-0.22 (-0.73, 0.30)

**Table 3:** Incidence of cutaneous melanoma, lung and bronchus, and breast changes among non-Hispanic White adult women in the U.S. (2001-2019) Nevada and Mississippi were excluded from the analysis because data were not available. Oklahoma did not report cutaneous melanoma data. SEER*Stat 8.3.9.2 and the Joinpoint Regression Program 4.9.0.0 were used to calculate cancer incidences and trends per 100,000. Statistically significant results are bolded for ease of reference. ᵃ Incidences reported per 100,000 AAPC, average annual percent change Source: United States Cancer Statistics [17]

States	AAPCᵃ (95% CI)	P-value
Melanoma		
High climate impact		
Arkansas	4.82 (4.04, 5.60)	<0.001
Utah	4.23 (3.55, 4.90)	<0.001
North Carolina	3.64 (2.06, 5.24)	<0.001
Missouri*	2.99 (1.14, 4.86)	0.001
Florida	2.78 (2.28, 3.28)	<0.001
Kansas	2.23 (1.44, 3.03)	<0.001
California	1.72 (1.42, 2.03)	<0.001
Moderate climate impact		
North Dakota	5.44 (2.39, 8.59)	<0.001
South Dakota	4.92 (3.68, 6.19)	<0.001
Nebraska	4.68 (1.87, 7.58)	0.001
Wisconsin	2.85 (2.14, 3.58)	<0.001
Idaho	2.09 (0.90, 3.29)	0.002
West Virginia	1.55 (0.69, 2.41)	0.001
New Jersey	0.81 (0.13, 1.48)	<0.001
Low climate impact		
Minnesota	4.56 (3.92, 5.21)	<0.001
Illinois	4.10 (3.49, 4.71)	<0.001
Wyoming	1.92 (0.52, 3.33)	0.01
Vermont	1.80 (0.48, 3.14)	0.01
Maine	1.74 (0.87, 2.62)	0.001
Pennsylvania	1.20 (0.18, 2.23)	0.021
Lung and bronchus		
High climate impact		
Arkansas	0.32 (0.06, 0.58)	0.02
Georgia	-0.41 (-0.76, -0.07)	0.02
Louisiana	-0.69 (-0.98, -0.40)	<0.001
Oklahoma	-0.91 (-1.32, -0.50)	<0.001
Florida	-1.03 (-1.28, -0.79)	<0.001
Texas	-1.22 (-1.68, -0.76)	<0.001
California	-1.99 (-2.26, -1.72)	<0.001
Moderate climate impact		
Tennessee	1.12 (0.36, 1.88)	0.004
South Dakota	0.98 (0.45, 1.52)	0.001
New Jersey	-0.56 (-0.88, -0.23)	0.002
Arizona	-1.34 (-1.87, -0.80)	<0.001
New Mexico	-1.61 (-2.22, -0.99)	<0.001
Oregon	-2.07 (-2.79, -1.34)	<0.001
District of Columbia	-2.86 (-4.72, -0.96)	0.006
Low climate impact		
Michigan	-0.46 (-0.91, 0.00)	0.048
Wyoming	-0.87 (-1.69, -0.04)	0.041
Colorado	-1.40 (-1.74, -1.06)	<0.001
Hawaii	-1.96 (-3.14, -0.76)	0.003
Breast		
High climate impact		
Florida	-0.52 (-0.80, -0.24)	<0.001
Moderate climate impact		
Delaware	0.66 (0.05, 1.27)	0.036
North Dakota	0.59 (0.15, 1.03)	0.012
Tennessee	0.40 (0.18, 0.63)	0.002
New York	0.38 (0.20, 0.57)	<0.001
Virginia	0.24 (0.01, 0.47)	0.042
District of Columbia	-1.54 (-2.85, -0.22)	0.025
Low climate impact		
Rhode Island	0.62 (0.22, 1.02)	0.004
New Hampshire	0.44 (0.05, 0.84)	0.03
Alaska	-0.77 (-1.21, -0.32)	0.002

**Table 4:** Incidence of cutaneous melanoma, lung and bronchus, and breast changes among non-Hispanic Black adult women in the U.S. (2001-2019) Nevada and Mississippi were excluded from the analysis because data were not available. Oklahoma did not report cutaneous melanoma data. SEER*Stat 8.3.9.2 and the Joinpoint Regression Program 4.9.0.0 were used to calculate cancer incidences and trends per 100,000. Statistically significant results are bolded for ease of reference. ᵃ Incidences reported per 100,000 AAPC, average annual percent change Source: United States Cancer Statistics [17]

States	AAPCᵃ (95% CI)	P-value
Melanoma		
High climate impact		
Florida	-2.33 (-4.20, -0.42)	0.02
Moderate climate impact		
Maryland	-2.25 (-4.34, -0.11)	0.041
Tennessee	-4.02 (-7.43, -0.48)	0.029
Low climate impact		
Illinois	-2.67 (-4.88, -0.41)	0.023
Lung and bronchus		
High climate impact		
Arkansas	11.36 (0.18, 2.56)	0.026
Georgia	-0.39 (-0.76, -0.02)	0.039
Louisiana	-0.82 (-1.39, -0.26)	0.004
Texas	-1.50 (-1.93, -1.07)	<0.001
Florida	-1.56 (-1.96, -1.17)	<0.001
Moderate climate impact		
Maryland	-0.99 (-1.53, -0.45)	0.001
Indiana	-1.08 (-1.81, -0.35)	0.006
New Jersey	-1.36 (-1.87, -0.85)	<0.001
Washington	-1.62 (-2.51, -0.73)	0.001
Arizona	-2.36 (-3.84, -0.85)	0.004
Low climate impact		
Pennsylvania	-0.68 (-1.05, -0.31)	0.001
Michigan	-1.31 (-1.81, -0.81)	<0.001
Massachusetts	-1.34 (-2.39, -0.27)	0.017
Colorado	-2.01 (-3.46, -0.54)	0.011
Rhode Island	-3.36 (-6.12, -0.52)	0.023
Breast		
High climate impact		
Alabama	1.04 (0.53, 1.56)	<0.001
Arkansas	1.02 (0.47, 1.56)	0.001
North Carolina	0.96 (0.67, 1.26)	<0.001
Georgia	0.90 (0.63, 1.17)	<0.001
South Carolina	0.83 (0.48, 1.19)	<0.001
Louisiana	0.73 (0.43, 1.04)	<0.001
Moderate climate impact		
New York	1.27 (1.01, 1.52)	<0.001
New Jersey	0.96 (0.52, 1.40)	<0.001
Connecticut	0.80 (0.23, 1.38)	0.009
Virginia	0.63 (0.20, 1.07)	0.007
Ohio	0.49 (0.14, 0.85)	0.009

**Table 5:** Incidence of cutaneous melanoma, lung and bronchus, and breast changes among Hispanic adult women in the U.S. (2001-2019) Nevada and Mississippi were excluded from the analysis because data were not available. Oklahoma did not report cutaneous melanoma data. SEER*Stat 8.3.9.2 and the Joinpoint Regression Program 4.9.0.0 were used to calculate cancer incidences and trends per 100,000. Statistically significant results are bolded for ease of reference. ᵃ Incidences reported per 100,000 AAPC, average annual percent change Source: United States Cancer Statistics [17]

States	AAPCᵃ (95% CI)	P-value
Melanoma		
High climate impact		
California	0.89 (0.10, 1.69)	0.029
South Carolina	-5.77 (-11.16, -0.05)	0.048
Moderate climate impact		
Arizona	3.83 (1.63, 6.08)	0.002
New Jersey	-1.95 (-3.65, -0.22)	0.03
Ohio	-4.81 (-9.36, -0.04)	0.048
Low climate impact		
Illinois	2.96 (1.05, 4.90)	0.004
Colorado	2.04 (0.34, 3.77)	0.021
Lung and bronchus		
High climate impact		
Georgia	3.25 (0.75, 5.80)	0.014
California	-1.32 (-1.75, -0.89)	<0.001
Texas	-2.24 (-2.61, -1.86)	<0.001
Louisiana	-2.36 (-4.50, -0.17)	0.036
South Carolina	-6.78 (-9.56, -3.91)	<0.001
Moderate climate impact		
New York	0.55 (0.01, 1.08)	0.046
Kentucky	-5.99 (-10.52, -1.23)	0.017
Low climate impact		
Hawaii	3.53 (0.85, 6.29)	0.012
Breast		
High climate impact		
Arkansas	2.38 (0.54, 4.26)	0.014
Georgia	2.29 (1.12, 3.47)	0.001
California	0.65 (0.44, 0.86)	<0.001
Moderate climate impact		
Tennessee	2.38 (0.67, 4.12)	0.009
Maryland	1.38 (0.79, 1.97)	<0.001
Indiana	1.31 (0.56, 2.07)	0.002
New Mexico	0.92 (0.36, 1.47)	0.003
New York	0.90 (0.59, 1.21)	<0.001
New Jersey	0.77 (0.18, 1.36)	0.014
Low climate impact		
Rhode Island	1.50 (0.24, 2.78)	0.022
Illinois	1.16 (0.60, 1.71)	<0.001
Colorado	0.70 (0.15, 1.26)	0.016
Michigan	-2.62 (-3.49, -1.73)	<0.001

**Table 6:** Incidence of Cutaneous melanoma, lung and bronchus, and breast changes among non-Hispanic Asian adult women in the U.S. (2001-2019) Nevada and Mississippi were excluded from the analysis because data were not available. Oklahoma did not report cutaneous melanoma data. SEER*Stat 8.3.9.2 and the Joinpoint Regression Program 4.9.0.0 were used to calculate cancer incidences and trends per 100,000. Statistically significant results are bolded for ease of reference. ᵃ Incidences reported per 100,000 AAPC, average annual percent change Source: United States Cancer Statistics [17]

States	AAPCᵃ (95% CI)	P-value
Melanoma		
Low climate impact		
Hawaii	-2.88 (-5.26, -0.44)	0.024
Lung and bronchus		
High climate impact		
Texas	-1.51 (-2.74, -0.27)	0.02
Moderate climate impact		
New York	2.65 (1.92, 3.39)	<0.001
Low climate impact		
Minnesota	3.60 (1.43, 5.81)	0.003
Pennsylvania	1.56 (0.42, 2.72)	0.01
Breast		
High climate impact		
Louisiana	3.60 (1.65, 5.59)	0.001
Missouri	3.11 (2.04, 4.19)	<0.001
Georgia	2.33 (1.31, 3.37)	<0.001
North Carolina	2.21 (0.83, 3.61)	0.003
Texas	1.87 (1.35, 2.38)	<0.001
California	1.13 (0.88, 1.38)	<0.001
Moderate climate impact		
Iowa	4.63 (2.41, 6.90)	<0.001
Idaho	2.81 (0.29, 5.40)	0.031
Maryland	2.55 (1.45, 3.66)	<0.001
Indiana	2.08 (0.35, 3.84)	0.021
Ohio	1.73 (0.49, 2.98)	0.009
Washington	1.29 (0.52, 2.07)	0.003
Virginia	0.76 (0.15, 1.37)	0.018
Low climate impact		
Rhode Island	4.29 (1.18, 7.49)	0.022
Illinois	2.01 (1.58, 2.45)	<0.001
Colorado	1.89 (0.73, 3.06)	0.016
Michigan	1.39 (0.30, 2.50)	<0.001

## Discussion

Our analysis identified statistically significant associations between state-level climate impact categories and cancer incidence trends across three cancer types among U.S. women from 2001 to 2019. Specifically, we observed differential incidence patterns in cutaneous melanoma, lung cancer, and breast cancer across high, moderate, and low climate impact states. Notably, racial disparities in incidence trends were evident, with racial/ethnic minorities experiencing greater increases in melanoma and breast cancer incidence in high climate impact regions. This ecological-level analysis provides a foundation for generating hypotheses about potential climate-health associations.

Our study found that high climate impact states reported greater melanoma incidences when compared to low climate impact states. These findings suggest that factors related to climate change may contribute to these cancer incidence trends. Prior research indicates that increased UV radiation exposure may contribute to melanoma risk [28]. Furthermore, warming temperatures and changes in outdoor recreation patterns could be among several factors that potentially influence melanoma incidence in high climate impact regions [29-31]. Additionally, higher temperatures during winter and spring seasons encourage people to stay outdoors longer, leading to a higher received UV dosage, which leads to a potentially greater risk of skin cancer [32].

White populations demonstrated the highest melanoma incidence increases across all climate impact categories. The differential susceptibility to UV radiation documented in dermatological literature may partially explain racial differences [33]. Moreover, the rising popularity of tanning salons, coupled with increased environmental UV radiation exposure, may exacerbate the risks of developing melanoma among White populations [34]. However, our study did not specifically capture such individual-level factors (e.g., tanning salon use, sunscreen practices, screening patterns, or residential UV exposure variability), which likely contribute substantially to observed incidence patterns.

Lung cancer incidence declined overall across all climate impact categories, consistent with historical trends reflecting decreased smoking prevalence. However, the magnitude of decline differed by climate impact category, with the smallest decline in high climate impact states versus larger declines in low impact states. Shifting weather patterns due to climate change can cause prolonged droughts and decreased precipitation, which lead to more frequent, severe, and widespread fires [35]. Prior reports indicate that these wildfires are associated with increased air pollution, and wildfire events and decreased air quality occur more frequently in high climate impact states [36-38]. As such, climate change-related environmental factors such as wildfire smoke and air pollution have been documented as potential cancer risk factors in other studies [38-41].

The slightly attenuated lung cancer decline in high climate impact states could reflect these environmental exposures, though our ecological design cannot establish causation. Racial disparities were notable, with the smallest declines among Black women in high climate impact states, in contrast to larger declines among Hispanic and Asian women. These disparities may reflect differences in environmental exposures or healthcare access, though this current study is limited in determining the specific factors with which these patterns are driven by. Possible factors include an unequal exposure to air pollution due to systematic discrimination in redlining and neighborhood segregation [42]. Prior work has shown that socioeconomically disadvantaged neighborhoods were associated with an increased lung cancer diagnosis [42]. On the other hand, it is conceivable that the racial and socioeconomic disparity in lung cancer risk may be due to differences in occupational exposure to harmful particles. It could potentially be further exacerbated by climate change-associated air pollution. This combination could be associated with increased lung cancer risks with outdoor occupations, such as agricultural workers, who are more directly exposed to extreme heat and smoke from wildfires, but further investigation is warranted [38,43].

Breast cancer incidence showed modest increases in high- and moderate-impact states but decreased in low-impact states. Ambient nitrogen dioxide and particulate matter 10 concentrations can cause deleterious genetic mutations that increase breast density and cancer risk [44]. Moreover, rising temperatures are associated with dose-dependent responses to increased absorption of these pollutants into the bloodstream that further increase breast cancer risk [44,45]. In addition, it is possible that temperature changes influenced by climate change may affect specific metabolic responses to EDCs, as studies have shown that the increasing ambient temperature has made model organisms more susceptible to cancer risks [46]. Higher temperatures can increase estrogen runoff, and synthetic estrogen also potentially contributes to increasing breast cancer incidences in high climate impact states [47]. Increased estrogen exposure has been associated with breast cancer in women, potentially through the proliferation of breast epithelial tissues [48,49]. Significant levels of environmental estrogen found in drinking, sewage, and irrigation water can be potentially made more harmful by the increasing temperature from climate change [50,51]. Additionally, heat stress is associated with decreased estrogen levels [51]. Of note, correlations between breast cancer and environmental estrogen as a byproduct of climate have not been clearly established in humans, and future research should be aimed at correlating data on estrogen levels with extreme weather events and human health.

Climate change-related extreme weather events can be associated with disrupted cancer screening facilities and oncology treatment centers that disproportionately affect disadvantaged communities. For instance, Hurricane Katrina impeded access to cancer care by disrupting transportation and depleting resources for accessing necessary healthcare [52]. Black residents in New Orleans accessed city infrastructures, including healthcare, much more slowly than White residents after Hurricane Katrina due to more severe housing damage from historic redlining practices [53]. These disparities in structural climate change recovery between racial groups could lead to a disproportionate increase in cancer incidences among racial minorities. Our study found that Hispanic, Asian, and Black populations had statistically significant increases in breast cancer incidence in high-impact regions, while the White population had no change. Specifically in regions with a high climate change impact, breast cancer incidence decreased for White populations but increased for all other populations. This striking divergence by race and ethnicity within the same climate category suggests that factors beyond climate-specific exposures (e.g., structural inequities, healthcare access disparities, or sociodemographic factors) may substantially influence these patterns. Environmental disparities arising from historical redlining and neighborhood segregation are well-documented [42], and such structural factors may interact with environmental exposures to compound and produce the observed incidence disparities.

Our study employs an ecological design with important methodological limitations that readers should consider when interpreting findings. Ecological fallacy, the risk of drawing individual-level inferences from population-level data, represents a fundamental constraint. We categorized climate exposure at the state level, which creates potential for substantial within-state exposure heterogeneity and misclassification of individual exposure. Additionally, temporal differences misalignment latency period for cancer development typically spans years to decades, while our climate categorization reflects 2007-2023 data patterns that may not align with exposure periods during relevant etiological windows (potentially 10-30 years prior to diagnosis). Moreover, this study did not formally model lag effects between climate exposures and cancer incidence given the ecological nature of the analysis and the absence of individual-level exposure timing data. Sensitivity analyses using alternative weighting schemes (e.g., prioritizing federal sources, weighting by temporal coverage, or excluding insurance-based metrics) were not performed in this study due to the exploratory nature of the analysis and the absence of an established weighting framework.

Another limitation of this study is that data available from USCS is limited, and specific data on demographic information (e.g., socioeconomic status, income, occupation, education, diet, trends in health screening and care, and other lifestyle factors), other environmental risk factors (e.g., UV index, water quality, air pollution, PFAS and microplastic pollution), and non-modifiable risk factors (family history, genetic risk, and hormonal factors) were unavailable. Additionally, data on cultural differences between U.S. states, such as diet and physical activity, that could potentially play a role in cancer development were also unavailable. As such, analyses that could adjust for these variables at the patient and population level could not be carried out to further assess the relationship between climate vulnerability and cancer trends. The state-level patterns for each unique extreme weather event were also not examined. We acknowledge that broader structural and behavioral factors, such as differences in screening practices, migration patterns, population aging, and regional variations in cancer registry completeness, may further interact with these unmeasured confounders and contribute to the observed incidence patterns. Due to the aforementioned limitations, the study does not conclude causational relationships between climate patterns and cancer incidences but rather a significant correlation between the variables and should not solely be interpreted as evidence of direct causal relationships between climate factors and cancer incidence. Therefore, the study identifies correlations between state-level climate-impact categories and cancer incidence trends and does not establish direct causation.

Despite its limitations, our data found statistically significant associated trends and incidences of three specific climate change-related cancers by states with varying climate vulnerability and impact. Over a 19-year period study, we utilize national-level and statewide data, making the results generalizable to U.S. women. Furthermore, the available racial data highlights the disproportionate impacts of climate change between racial groups, which highlights the importance of caring for minority populations in climate change-impacted states. This study provides one of the few national-level examinations of how cancer incidence patterns among U.S. women align with varying degrees of climate vulnerability, offering an early but important foundation for understanding emerging environmental health disparities and guiding future, more detailed investigations. The pronounced disparities in breast cancer and melanoma incidence by race and ethnicity within high climate impact states represent a novel and significant empirical finding that warrants further investigation. The pronounced disparities in breast cancer and melanoma incidence by race and ethnicity within high climate impact states represent a novel and significant empirical finding that warrants further investigation.

## Conclusions

Our results indicate that cancer incidence patterns among U.S. women differ across climate-impact categories and racial groups, suggesting that climate vulnerability may be associated with persistent disparities in melanoma, lung, and breast cancer trends. While these findings are ecological in nature, they provide an important foundation for future studies incorporating individual-level data, confounding adjustment, and granularized climate metrics. As climate-related events intensify, such work will be essential for guiding evidence-based policies aimed at reducing environmental health inequities.
